# Pyroglutamyl leucine, a peptide in fermented foods, attenuates dysbiosis by increasing host antimicrobial peptide

**DOI:** 10.1038/s41538-019-0050-z

**Published:** 2019-10-07

**Authors:** Saki Shirako, Yumi Kojima, Naohiro Tomari, Yasushi Nakamura, Yasuki Matsumura, Kaori Ikeda, Nobuya Inagaki, Kenji Sato

**Affiliations:** 1 Division of Applied Biosciences, Graduate School of Agriculture, Kyoto University, Kitashirakawa Oiwake-cho, Kyoto 606 8502 Japan; 2 Kyoto Municipal Institute of Industrial Technology and Culture, 91 Chudouji Awata-cho, Kyoto, 600 8813 Japan; 3 Department of Japanese Food Culture, Kyoto Prefectural University, Shimogamo-Hangi-cho, Kyoto, 606 8522 Japan; 4 Division of Agronomy and Horticultural Science, Graduate School of Agriculture, Kyoto University, Gokasho, Uji, Kyoto, 611 0011 Japan; 5 Department of Diabetes, Endocrinology and Nutrition, Graduate School of Medicine, Kyoto University, 54 Shogoin Kawahara-cho, Kyoto, 606 8507 Japan

**Keywords:** Peptides, Nutrition

## Abstract

PyroGlu-Leu is present in certain food protein hydrolysates and traditional Japanese fermented foods. Our previous study demonstrated that the oral administration of pyroGlu-Leu (0.1 mg/kg body weight) attenuates dysbiosis in mice with experimental colitis. The objective of this study was to elucidate why such a low dose of pyroGlu-Leu attenuates dysbiosis in different animal models. High fat diet extensively increased the ratio of *Firmicutes/Bacteroidetes* in feces of rats compared to control diet. Oral administration of pyroGlu-Leu (1 mg/kg body weight) significantly attenuated high fat diet-induced dysbiosis. By focusing on the production of intestinal antimicrobial peptides, we found that pyroGlu-Leu significantly increased the level of 4962 Da peptides, which identified as the propeptide of rattusin or defensin alpha 9, in ileum. We also observed increased tryptic fragment peptides from rattusin in the lumen. Here, we report that orally administered pyroGlu-Leu attenuates dysbiosis by increasing in the host antimicrobial peptide, rattusin.

## Introduction

Food peptides are frequently produced by protease digestion of food proteins. Furthermore, peptides are contained in certain fermented foods. Although it was initially assumed that all the peptides found in food are degraded into amino acids during the digestion and absorption processes, it has been demonstrated that some peptides can resist protease digestion and can be absorbed directly into the blood.^1,2^ The peptides in foods have been found to have biological functions in addition to being sources of amino acids.^3,4^ Food protein hydrolysates and fermented foods contain pyroglutamyl peptides, which are spontaneously generated from peptides with a glutaminyl residue at the amino terminal during storage and processing.^5–7^ Short chain pyroglutamyl peptides are resistant to digestion by endoproteinases and exopeptidases. Some of the short chain pyroglutamyl peptides have been demonstrated to have in vivo^8–11^ and in vitro^12,13^ activities. Studies have shown that the oral administration of pyroglutamyl leucine (pyroGlu-Leu or pEL), which was initially identified in wheat gluten hydrolysate, attenuates hepatitis^8^ and colitis^9^ in animal models. A latter study revealed that the oral administration of very low doses (0.1–1.0 mg/kg body weight) of pyroGlu-Leu can normalize the disturbances in the colonic microbiota of mice with dextran sulfate sodium (DSS)-induced colitis.^9^ The pathological disturbance of gut microbiota is referred to as dysbiosis.^14^ It was seen that another related pyroglutamyl peptide, pyroGlu-Asn-Ile, also ameliorated DSS-induced dysbiosis in mice at 1.0 mg/kg body weight.^11^ PyroGlu-Leu is also present in the enzymatic hydrolysates of corn gluten and fish, as well as in certain traditional Japanese fermented foods.^15^

It has been previously reported that certain foods and food components can improve the gut microbiota.^16,17^ In many cases, foods containing live microorganisms that have beneficial effects on the host or those with nutrients that are beneficial for the gut microorganisms, referred to as probiotics and prebiotics, respectively, are used for their health benefits as they directly target microorganisms found in the gut. An effective dose of probiotics and prebiotics is around 100 mg–1.0 g/kg body weight.^16,17^ Alternatively, the oral administration of lactoferrin, which produces naturally occurring antimicrobial peptides, such as lactoferricin, by peptic digestion, has been demonstrated to modulate the gut microbiota.^18^ However, in our previous study, we demonstrated that pyroGlu-Leu attenuates DSS-induced dysbiosis at a dose of 0.1 mg/kg body weight without increasing the level of pyroGlu-Leu in the colon. Therefore, it is unlikely that pyroGlu-Leu, at such small doses, can directly enhance and suppress the growth of microorganisms in the colon. On the other hand, the epithelial surfaces of tissues from organs such as the intestine, skin, and respiratory and reproductive tracts, secrete antimicrobial peptides.^19^ These antimicrobial peptides exert their bactericidal activity mainly by damaging the cell wall of the bacteria.^19^ Peptides such as α-defensins, rattusin (α-defensin-related peptide), lysozyme, REG3, and cathelicidins are secreted into the lumen from the intestine to suppress the growth of bacteria in the small intestine.^19^ Guo et al. demonstrated that the expression of intestinal antimicrobial peptides in mice with high fat diet-induced colonic dysbiosis was lower than that in normal mice.^20^ Furthermore, it has been demonstrated that the secretion of α-defensin 5 in the small intestine is relatively suppressed in obese human subjects with dysbiosis compared to normal subjects.^21^ Based on these findings, it is speculated that intestinal antimicrobial peptides might control colonic microbiota; hence, a decrease in the intestinal antimicrobial peptides can induce dysbiosis. Indeed, the oral administration of mouse α-defensin (cryptdin-4) improves graft-versus-host disease-mediated dysbiosis.^22^ Therefore, food components capable of enhancing the production of host antimicrobial peptides in the intestine can also improve colonic microbiota.

These findings suggest that the administration of low doses of pyroGlu-Leu may attenuate dysbiosis by enhancing the production of host antimicrobial peptides. The present study aimed to elucidate the effects of pyroGlu-Leu on the production of intestinal antimicrobial peptides using rats with high fat diet-induced dysbiosis.

## Results

### Effect of pyroGlu-Leu on high fat diet-induced dysbiosis

**Fig. 1:**
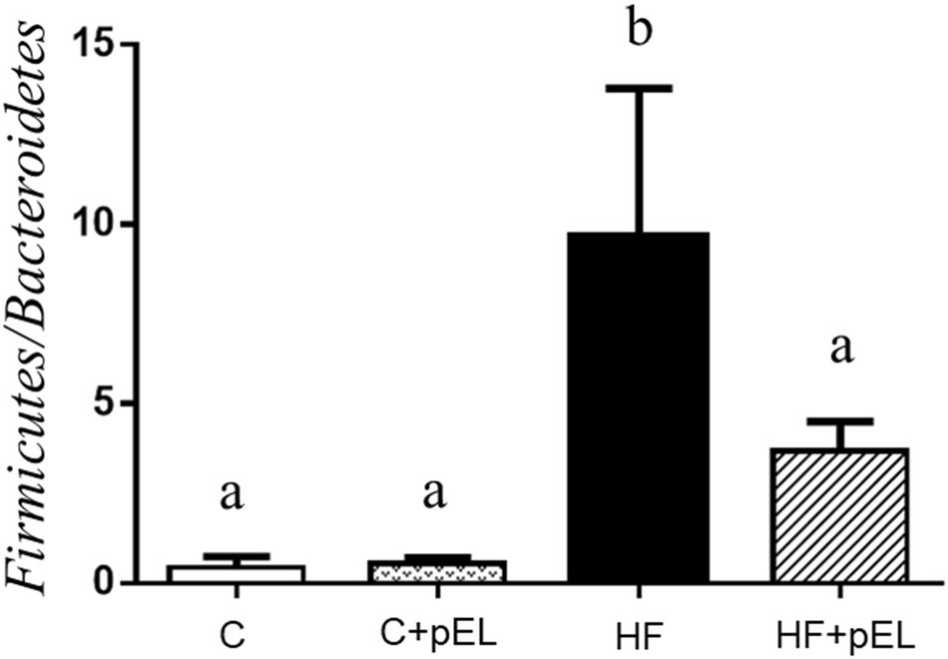
Effect of administration of pyroGlu-Leu (pEL) on the ratio of *Firmicutes* to *Bacteroidetes* in the feces. Control group (C), control + pEL group (C + pEL), high fat diet group (HF), high fat diet + pEL group (HF + pEL), respectively. The *Y* axis represents the ratio of *Firmicutes*/*Bacteroidetes*. Different letters indicate significant differences (*p* < 0.05) by Tukey’s test (*n* = 3). The results are presented as the mean ± SD

As shown in Fig. 1, rats on a high fat diet (HF) without pyroGlu-Leu administration displayed increased *Firmicutes/Bacteroidetes* ratio in their feces compared to the rats in the control diet groups (C and C + pEL; *p* = 0.003 by Tukey’s test, *n* = 3), which indicates that dysbiosis was induced by a high fat diet. The administration of pyroGlu-Leu (1.0 mg/kg body weight) significantly attenuated high fat diet-induced dysbiosis (HF + pEL; *p* = 0.035 by Tukey’s test, *n* = 3). The administration of pyroGlu-Leu did not affect the *Firmicutes/Bacteroidetes* ratio in the control diet groups (C and C + pEL; *p* > 0.99 by Tukey’s test, *n* = 3). However, the administration of pyroGlu-Leu did not significantly affect body weight gain and blood biochemical parameters (ALT, AST, TCHO, TG, HDL, and LDL) in the rats in the high fat diet groups (*p* > 0.05 by Tukey’s test, *n* = 3: Data not shown).

### Comprehensive analysis of peptides in 30% acetic acid extracts

The peptides extracted using 30% acetic acid from the ileums of rats (*n* = 3) from 4 experimental groups (C, C + pEL, HF, and HF + pEL) were fractionated using size exclusion chromatography (SEC). The peptides in the SEC fractions were resolved by reversed phase-HPLC (RP-HPLC). The elution of peptides by RP-HPLC was monitored by electron spray ionization mass spectrometry (ESI-MS) in total ion monitoring (TIM) mode. Sixty-nine peaks were observed in the total ion chromatograms of RP-HPLC of the SEC fractions from the rats (*n* = 3) in each group (Supplemental Fig. 1a–d). Table 1 summarizes the retention time and observed mass-to-charge ratio (*m/z*). The first peak (peak 1) appeared in SEC fractions 14–26 and 30–35, respectively. The peptides in the former and latter SEC fractions showed the same retention time and mass spectrum pattern in RP-HPLC-MS, which suggests that the peptide in peak 1 in the former SEC fraction forms an oligomer via non-covalent bonding. Based on the *m/z* of the multivalent ions, the molecular weights of 57 peptides were estimated (Table 1).

**Table 1:** Summary of the peaks obtained by the LC-MS of the SEC fractions

Peak no.	SEC Fr.	RT	Observed *m/z*	Estimated molecular weight	SIM
1	14-20, 32-34 (C), 14-21, 32-33 (CP), 14-26, 31-34 (HF), 14-26, 30-35 (HP)	11.2	723.7, 761.8, 804.0, 851.2, 904.4, 964.6, 1033.4, 1112.9, 1205.4, 1315.0, 1446.4, 1607.0	14453	○
2	17-18 (C)	11.8	775.7, 816.6, 861.8, 912.5, 969.4, 1034.0, 1107.7, 1192.9, 1292.2	15493	○
3	17-18 (C), 15-19 (HF), 14-19 (HP)	11.7	796.2, 843.0, 895.6, 955.2, 1023.4, 1102.1, 1193.8, 1302.3, 1432.3, 1591.5	14312	○
4	22-27 (C), 21-25 (CP), 21-24 (HF), 21-24 (HP)	10.5	879.9, 932.6, 989.7, 1055.6, 1131.0, 1217.7, 1319.2	15825	○
5	14-16 (CP)	14.6	532.4, 576.4, 620.5	―	―
6	20, 23 (CP)	12.8	787.2, 847.5, 885.5, 917.8, 972.0	―	―
7	14-19 (HF), 14-20 (HP)	12.3	752.7, 792.2, 836.2, 885.3, 940.7, 1003.2, 1074.8, 1157.4, 1253.9, 1367.6, 1504.5, 1671.5	15033	○
8	30-32 (C), 29-31 (CP), 29-32 (HF), 29-31 (HP)	10.7	739.9, 778.5, 821.9, 870.2, 924.5, 986.1, 1056.5, 1137.7	14779	○
9	30-33 (C), 29-31 (CP), 29-31 (HF), 29-31 (HP)	11.4	721.8, 773.3, 832.6, 902.0, 983.9	10812	○
10	30-31 (C), 29-31 (CP), 28-31 (HF), 28-30 (HP)	11.1	814.5, 862.3, 916.1, 977.1, 1046.9	14644	○
11	31 (C)	11.3	782.4, 831.2, 886.5, 949.8, 1022.7	13289	○
12	33-35 (C), 34-35 (CP), 33-35 (HF), 33-35 (HP)	10.9	765.5, 829.1, 904.4, 994.7, 1105.2, 1243.1	9941	○
13	31 (C), 25-27 (CP), 26 (HF)	12.5	846.1, 890.2, 937.1, 989.0	17535	○
14	31-32 (C), 30-32 (CP), 30-32 (HF), 29-32 (HP)	12.2	807.5, 846.0, 888.1, 934.8, 986.7, 1044.6	17748	○
15	26-27 (C), 25-27 (CP), 25-27 (HF), 25-26 (HP)	9.2	973.7, 1459.8	2919	○
16	29-30 (C), 29-30 (CP), 28 (HF), 29-30 (HP)	7.8	696.9, 1045.1	2086	○
17	29-30 (C), 29-30 (CP), 29-30 (HF), 28-30 (HP)	8.7	749.3, 998.9	2991	○
18	28-30 (C), 28 (HF)	9.5	564.3, 868.1, 1166.8	―	―
19	30-33 (C), 29-33 (CP), 29-33 (HF), 28-33 (HP)	12.4	724.6, 760.8, 800.8, 845.3, 894.9, 950.8	15191	○
20	30-33 (C), 31-33 (CP), 31-33 (HF), 30-33 (HP)	8.3	621.3, 710.0, 828.1, 993.5	4962	○
21	31 (C), 29-31 (CP), 30-31 (HF)	7.5	555.1, 739.6	2218	○
22	31 (C), 31 (CP), 31 (HF)	7.2	537.0, 715.5, 1032.6	2234	○
23	31 (C)	8.5	536.9, 716.0	2139	N.D.
24	31-31 (C), 31-34 (CP), 30-34 (HF), 30-34 (HP)	13.3	754.9, 792.6, 834.3, 880.6, 932.4	15823	○
25	32-34 (C), 32-33 (CP), 32-33 (HF), 32-33 (HP)	12.0	726.0, 766.3, 811.3, 861.9	13777	○
26	32-33 (C), 32 (CP), 32 (HF), 31-32 (HP)	8.0	712.3, 830.8, 996.9	4978	○
27	33-34 (C), 33-34 (CP), 33 (HF), 33 (HP)	10.1	717.5, 789.2, 876.8, 986.3	7878	○
28	33-34 (C), 33-34 (CP), 33-34 (HF), 32-33 (HP)	6.1	771.4, 841.8, 925.7, 1028.7, 1156.9	9239	○
29	33-34 (C), 32-33 (CP), 33 (HP)	10.1	706.3, 734.5, 765.2, 798.4, 834.7, 874.3, 918.0	18330	○
30	33 (C), 33 (CP), 33 (HF), 33 (HP)	8.5	706.1, 759.9, 823.6	9872	○
31	34 (C)	13.4	895.3, 995.2, 1119.6, 1279.3	8931	○
32	34-35 (C), 35 (HF)	8.7	577.8, 720.6, 957.0, 1063.1, 1196.1	―	―
33	34-35 (C), 34-35 (CP), 33-35 (HF), 34-35 (HP)	7.4	566.9, 708.4, 944.0	2830	○
34	34 (C)	9.2	636.0, 684.2, 739.0, 769.7, 839.4, 923.4	―	―
35	34-35 (C), 34-35 (CP)	9.5	712.1, 745.8, 783.2, 824.3, 870.0	15653	N.D.
36	34-35 (C), 34-35 (CP), 34-35 (HF), 34 (HP)	12.2	701.3, 738.2, 779.1, 824.8	14013	○
37	35 (C)	9.8	736.1, 809.6, 899.4, 1011.6, 1156.0	8087	N.D.
38	25-26 (CP), 24-26 (HF), 25-27 (HP)	8.6	1019.1, 1273.4	5094	○
39	27 (CP)	8.9	674.9, 911.8	2590	○
40	28 (CP)	8.3	654.3, 980.9	1960	○
41	30-31 (CP), 30 (HF)	8.4	699.3, 932.3	2791	○
42	30 (CP)	10.1	644.3, 664.3, 685.7, 708.6, 732.9, 759.1	21240	N.D.
43	31 (CP), 31 (HF), 31 (HP)	6.8	695.6, 834.5	4168	○
44	31 (CP), 30 (HP)	10.4	942.1, 1046.7, 1177.4, 1345.5, 1569.1	9414	N.D.
45	32 (CP), 31-32 (HP)	9.0	681.8, 749.7, 833.0, 936.9, 1070.4, 1248.7	7491	○
46	33 (CP)	9.5	672.6, 691.9, 712.2, 733.8, 756.7	24138	N.D.
47	32 (CP)	10.3	828.5, 887.5, 935.2, 1168.9, 1335.9, 1580.0	―	―
48	33 (CP)	10.9	726.0, 760.7, 798.7, 840.6	15934	N.D.
49	34 (CP)	7.6	607.6, 708.8, 850.5, 1062.9	4244	○
50	34 (CP)	8.8	664.6, 691.4, 720.1, 751.4, 785.5, 822.8, 864.0	17232	N.D.
51	34 (CP)	7.7	626.4, 730.7, 876.8, 1095.4	4377	N.D.
52	34 (CP)	11.1	761.8, 821.4, 851.3, 904.4, 964.6, 1058.6, 1164.3, 1293.7	―	―
53	35 (CP), 35 (HF)	7.9	617.0, 649.7, 685.7, 725.8, 771.2, 822.4, 881.2	12314	N.D.
54	34-35 (CP), 35 (HF), 34-35 (HP)	13.5	841.9, 912.1, 994.9, 1094.2, 1215.7	10930	○
55	35 (CP), 35 (HF), 35 (HP)	12.9	889.2, 957.6, 1037.3, 1131.7, 1244.6, 1382.7	12433	○
56	28 (HF), 28 (HP)	9.2	520.3	520	○
57	29 (HF)	7.5	672.9, 779.5	4906	○
58	29-30 (HF)	7.8	697.0	697	○
59	29 (HF)	7.0	819.7, 983.8	4903	○
60	35 (HF)	8.1	524.9, 655.9, 1454.1, 1595.4, 1963.1	―	―
61	35 (HF), 35 (HP)	12.3	703.2, 739.4, 781.3, 827.1, 878.9, 937.4, 1003.3, 1157.3, 1671.6	―	―
62	29 (HP)	8.8	504.3, 1276.1	―	―
63	28 (HP)	14.5	532.4, 576.3, 620.4, 664.5, 708.4, 1425.5, 1613.9, 1828.1	―	―
64	29 (HP)	8.8	504.3, 648.9	2255	○
65	32 (HP)	10.6	765.0, 834.5, 917.9, 1019.7, 1147.1, 1310.8	9167	N.D.
66	33-34 (HP)	8.7	761.8, 888.5	5329	N.D.
67	34 (HP)	9.8	904.4, 1033.7, 1205.6	7225	N.D.
68	35 (HP)	6.4	549.7, 584.0, 603.0, 667.3, 718.8, 778.7, 849.2	―	―
69	35 (HP)	8.1	599.0, 718.4, 897.9	3588	○

The aliquots of the size exclusion chromatography fractions (SEC Fr.) were subjected to LC-MS

RT = retention time of LC-MS

The molecular weights were calculated using the observed multivalent ions

“―“ represents Molecular weight could not be calculated

“○”represents Peak was observed by selected ion monitoring (SIM) for the top 2 or 3 divalent ions.

“N.D.“ represents Peak could not be detected by SIM analysis

**Fig. 2:**
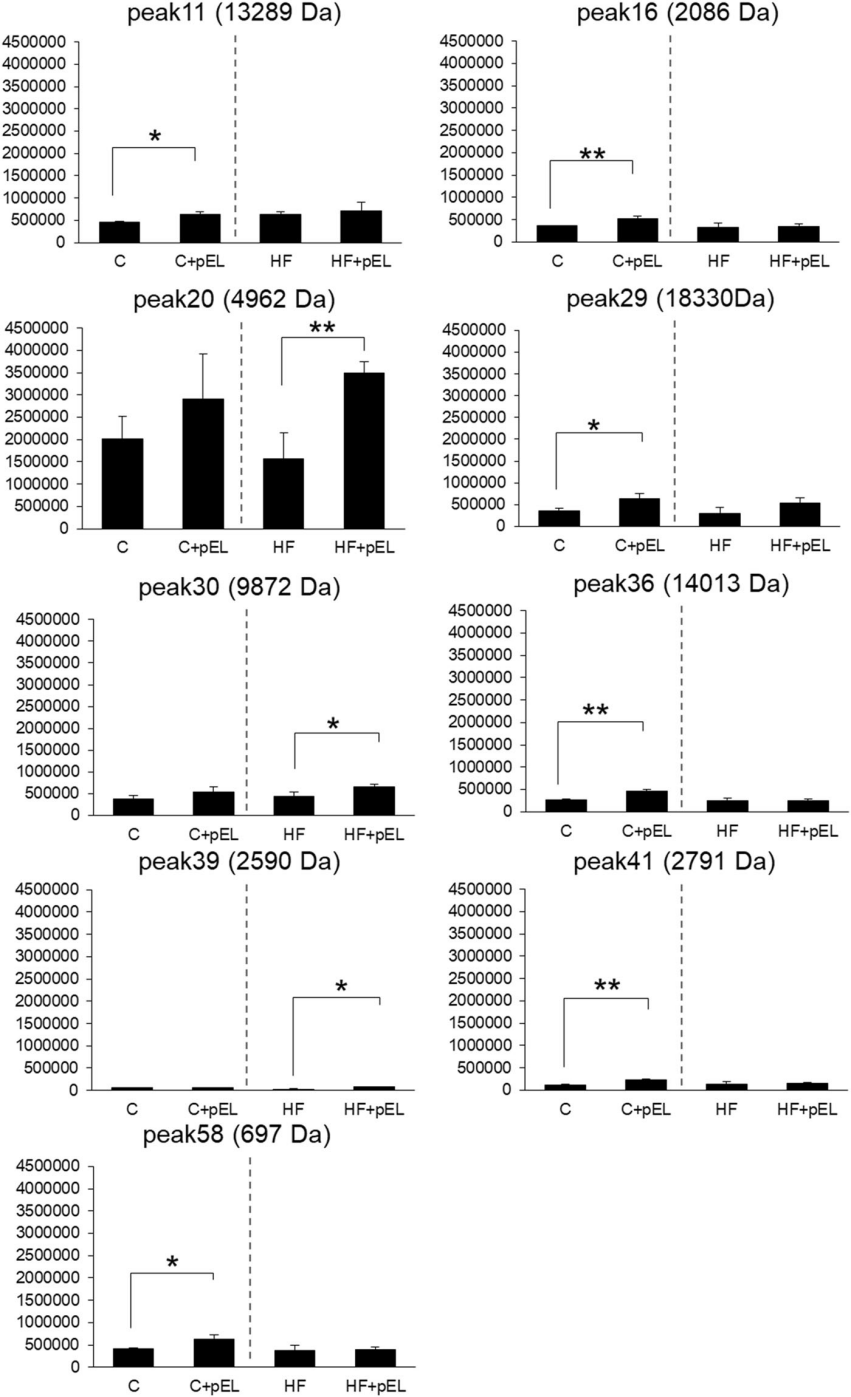
Effect of pyroGlu-Leu on the peak area of the peptides in the 30% acetic acid extract of ileum. Refer the figure legend of Fig. 1 for affiliation of animal groups. The Y axis represents peak area of LC-MS. Asterisks (**) and (*) represent *p* < 0.01 and *p* < 0.05, respectively, compared with the vehicle (C vs. C + pEL, HF vs. HF + pEL, respectively) by Student’s *t*-test (*n* = 3). The results are presented as the mean ± SD

The peptides in the 30% acetic acid extracts were directly detected using RP-HPLC-MS in selected ion monitoring (SIM) mode, as described in the Methods section. Forty-four peptides were detected. The level of 9 peptides were significantly changed after pyroGlu-Leu administration, as shown in Fig. 2 (*p* < 0.05 by *t*-test, *n* = 3). The peptides that were not significantly altered are shown in Supplemental Fig. 2 (*p* > 0.05 by *t*-test, *n* = 3). Among the control diet groups, the peptides in peaks 11 (13289 Da), 16 (2086 Da), 29 (18330 Da), 36 (14013 Da), 41 (2791 Da), and 58 (697 Da) significantly increased after the administration of pyroGlu-Leu (*p* = 0.02, 0.01, 0.04, 0.01, 0.01, and 0.02, respectively by *t*-test, *n* = 3). Among the high fat diet groups, the peptides in peaks 20 (4962 Da), 30 (9872 Da), and 39 (2590 Da) significantly increased after the administration of pyroGlu-Leu (*p* = 0.01, 0.04, and 0.03, respectively by *t*-test, *n* = 3). Among these three peptides, the peptide in peak 20 (4962 Da) showed the highest ion intensity. Further, the exact same molecular weight for the peptide in peak 20 was identified using matrix assisted laser desorption/ionization time of flight mass spectrometry (MALDI-TOFMS) analysis (Supplemental Fig. 3). This peptide was purified by RP-HPLC and subjected to amino acid sequence analysis. The resulting sequence (DPIEEAEEETKTE) correlated with the amino terminal region of the precursor form of the rat α-defensin-related peptide (rattusin) or defensin alpha 9 (_20_DPIQEAEEETKTE_32_), which are generated after the cleavage of the signaling peptide. These two antimicrobial peptides share the same propeptide amino terminal sequence, while the active regions differ in sequence. On the basis of the molecular weight, this peptide was identified as a propeptide of rattusin or defensin alpha 9 that is released after the conversion of its inactive precursor into the active form.

### Detection of peptides derived from the active form of rattusin and defensin alpha 9

**Fig. 3:**
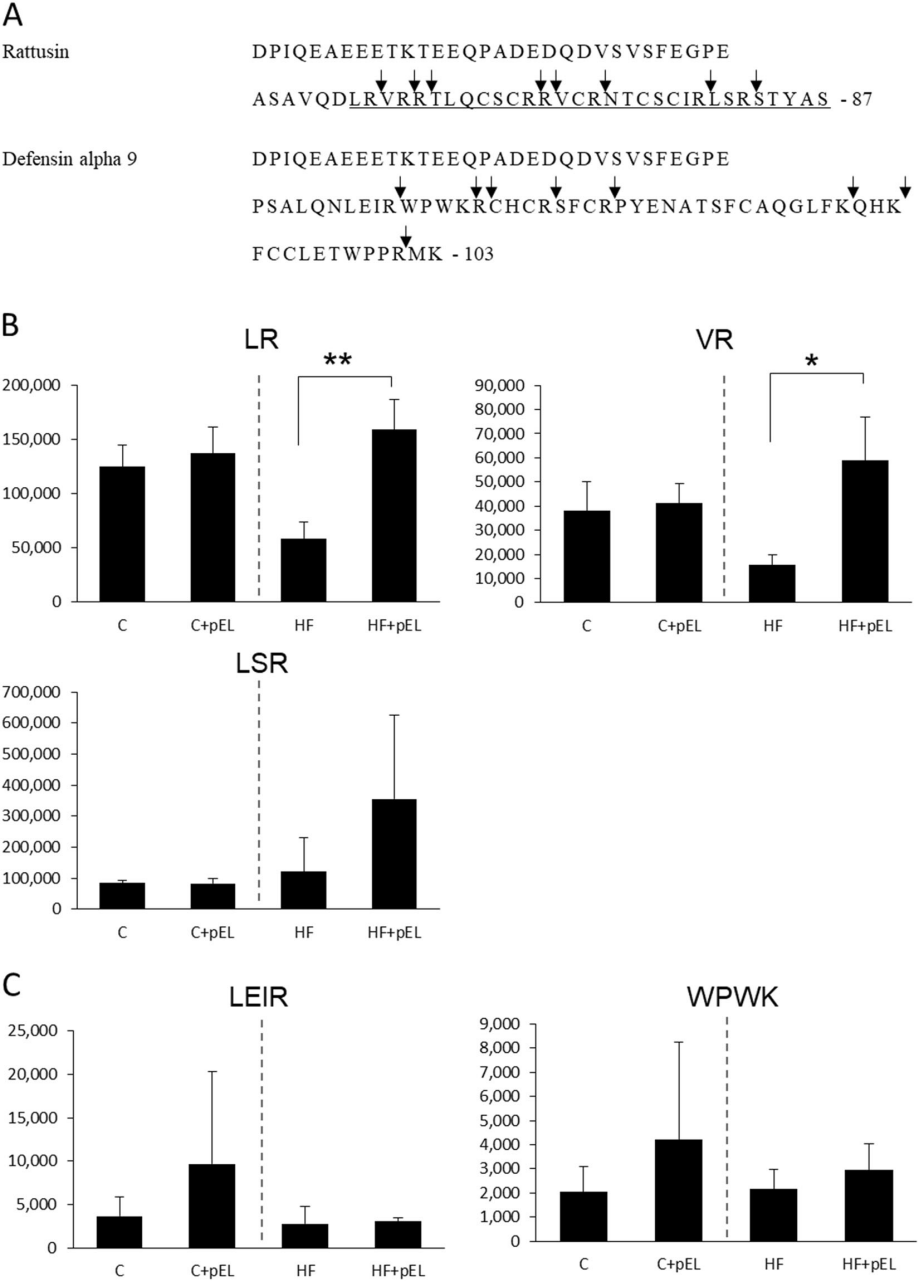
Detection of tryptic peptides from the active form of rattusin and defensin alpha 9. Refer the figure legend of Fig. 1 for affiliation of animal groups. The amino acid sequence of rattusin and defensin alpha 9 precursors, respectively. The sequence of known active form is underlined. Arrows indicate the trypsin cleavage sites **a**. Comparison of peak area of fragment peptides–rattusin **b** and defensin alpha 9 **c**. The *Y* axis represents peak area of LC-MS/MS. Asterisks (**) and (*) represent *p* < 0.01 and *p* < 0.05, respectively, compared with the vehicle (C vs. C + pEL, HF vs. HF + pEL, respectively) by Student’s *t*-test (*n* = 3). The results are presented as the mean ± SD

To detect the secretion of the active form of these antimicrobial peptides from the lumen, peptides from the inner contents of the small intestine were subjected to SEC and RP-HPLC-MS, as described in the Methods section. RP-HPLC-MS showed large broad peaks and mass spectra analysis revealed the presence of numerous compounds in these peaks. Therefore, it was difficult to identify the peptides in such complicated matrix based on sequence analysis. Alternatively, the peptides that were potentially released from the active form of rattusin and defensin alpha 9 via trypsin digestion were detected by LC-MS/MS in the multiple reaction monitoring (MRM) mode. The levels of rattusin-related peptides, LR, VR, and LSR, were found to be higher after pyroGlu-Leu administration in the high fat diet groups (Fig. 3, panels a and b), while this was not the case with the defensin alpha 9-related peptides, LEIR and WPWK, both in the control and high fat diet groups (Fig. 3, panels a and c).

## Discussion

Although antimicrobial peptides are usually detected by ELISA assay using specific antibodies, many types of antimicrobial peptides are present in the ileum. In addition, most of these antimicrobial peptides are synthesized in the precursor form and converted into their active form by the proteolytic removal of propeptides. To observe all the changes in the antimicrobial peptides in tissues, several kinds of ELISA systems are necessary. Antimicrobial peptides in the intestine can be extracted using a high concentration of acetic acid (final concentration of 30%).^23^ In the present study, a comprehensive analysis of peptides in 30% acetic acid extracts from the ileum was carried out. Using SEC and RP-HPLC in series along with ESI-MS analyses, 57 peptides with different molecular weights were detected. The administration of pyroGlu-Leu was found to significantly enhance the levels of the propeptide of rattusin or defensin alpha 9 in the rats fed with a high fat diet, suggesting that the oral administration of pyroGlu-Leu increases the production of the active form of rattusin or defensin alpha 9. However, we did not detect any increase in these peptides in their active forms either directly in the ileum extracts or the lumen using the same method. The active forms of antimicrobial peptides, such as α-defensins, exert their antimicrobial activity by perforating the cell walls of bacteria and can also potentially damage the host cells.^19^ To achieve this, these active peptides should be immediately excreted into the lumen in order to target the bacteria without damaging the host cells. Indeed, tryptic fragment peptides of rattusin were found in the lumen. In addition, we did not observe any significant decrease in the other peptides in the 30% acetic acid extract, which includes most of the antimicrobial peptides, to compensate for the increase of rattusin. These findings indicate that the oral administration of pyroGlu-Leu increases the excretion of the active form of rattusin into the lumen, which consequently suppresses excess proliferation of *Firmicute*s (Gram-positive bacteria) due to high fat diet as *Firmicutes* are more susceptible to certain antimicrobial peptides.^19^

Although the propeptide of rattusin, whose molecular weight is 4962 Da, could be detected in the 30% acetic acid extract from the ileum by direct injection to LC-MS in SIM mode, it was difficult to detect the active form of rattusin. Therefore, the propeptide of rattusin (4962 Da) can be used to monitor the activation of rattusin in rats to screen for food components that can enhance the production of rattusin. LC-MS and ELISA with the antibody against the propeptide of rattusin can be used for this purpose.

The mechanism underlying the enhancement of rattusin activation by pyroGlu-Leu remains to be elucidated. Intestinal α-defensins are known to be produced by the Paneth cells in the ileum.^24^ It has also been suggested that rattusin, which belongs to a defensin subfamily, is produced by Paneth cells.^25^ The level of rattusin propeptide in the ileum was found to be higher than that in duodenum and colon (Supplemental Fig. 4). Therefore, pyroGlu-Leu may interact directly with the Paneth cells in the ileum to produce rattusin. However, there is a possibility that pyroGlu-Leu interacts with other cells as well, such as macrophages and neutrophils, to enhance or suppress certain active substances that affect Paneth cells. To solve this problem, a cell culture system for rat Paneth cells and an intestinal organ culture system that produces rattusin are currently being developed.

To the best of our knowledge, there is no study demonstrating the enhancement of the production of host antimicrobial peptides by the oral administration of a single food component. It has been shown that pyroGlu-Leu is widely distributed in food protein hydrolysates, such as wheat gluten and corn gluten hydrolysates,^7^ as well as in Japanese fermented foods produced by *Asperigillus oryzae*, such as the Japanese rice wine known as sake, the salted fermented soy paste known as miso, and a type of soy sauce, shoyu.^15^ Fermented foods are consumed in the diet, while protein hydrolysates are generally consumed as supplement. Sake is not only consumed as an alcoholic beverage, but is also used as a seasoning in traditional Japanese dishes. Sake contains approximately 1.0–1.5 mg/100 mL of pyroGlu-Leu.^6^ Both miso and shoyu contain higher amounts of pyroGlu-Leu than sake.^15^ Therefore, significant amounts of pyroGlu-Leu can be obtained by the consumption of traditional Japanese foods resulting in the improvement of the gut microbiota due to the increased production of host antimicrobial peptides. Previous studies have suggested that the consumption of Japanese fermented foods may improve gastrointestinal conditions.^26^ The fiber and live microorganisms found in these fermented foods are thought to be responsible for their beneficial effect on the gut. In conclusion, in this study, we propose the concept that peptides produced during fermentation can improve the gut conditions by enhancing the host antimicrobial peptides. The initial findings reported need to be further validated by epidemiological studies and then confirmed by well-designed human clinical trials.

## Methods

### Reagents

*N*-(*tert*-Butoxycarbonyl)-L-pyroglutamic acid (Boc-Pyr-OH), L-leucine *tert*-butyl ester hydrochloride (H-Leu-OtBu・HCl) and acetonitorile (HPLC grade) were obtained from Wako Pure Chemical Industries (Osaka, Japan). *N*-α-(9-Fluorenylmethoxycarbonyl)-*N*-ω-(2,2,4,6,7-pentamethyldihydrobenzofuran-5-sulfonyl)-L-arginine *p*-methoxybenzyl alcohol resin (Fmoc-Arg(Pbf)-Alko Resin), Fmoc-L-Leu-OH, Fmoc-L-Val-OH, Fmoc-O-(*t*-butyl)-L-serine (Fmoc-Ser(*t*Bu)-OH), Fmoc-L-Ile-OH, and Fmoc-L-Trp-OH were obtained from Watanabe Chemical Industries (Hiroshima, Japan). Fmoc-*N*-ω-(*t*-butyloxycarbonyl)-L-lysine (Fmoc-Lsy(Boc)-Resin), Fmoc-L-Pro-OH, and Fmoc-L-glutamic acid γ-*t*-butyl ester (Fmoc-Glu(OtBu)-OH) were obtained from HiPep Laboratories (Kyoto, Japan).

### Peptides synthesis

PyroGlu-Leu (pEL) was synthesized using a manual lipid-phase method, as described previously.^8^ Tryptic digested peptides potentially released from the active form of rattusin (Leu-Arg, Val-Arg, and Leu-Ser-Arg) and defensin alpha 9 (Leu-Glu-Ile-Arg and Trp-Pro-Trp-Lys), except for the cysteine-containing peptides, were synthesized by the Fmoc strategy using an automatic peptide synthesizer (PSSM-8, Shimadzu, Kyoto, Japan). The synthesized peptides were purified by RP-HPLC using a Cosmosil MS-II (10 mm i.d. × 250 mm; Nacalai Tesque, Kyoto, Japan). The peptides were eluted with a binary gradient of 0.1% formic acid (solvent A) and 0.1% formic acid containing 80% acetonitrile (solvent B) at a flow rate of 2.0 mL/min. The gradient program was as follows: 0–20 min; B 0–50%, 20–30 min; B 50–100%, 30–35 min; B 100%, 35–35.1 min; B 100–0%, 35.1–45 min; B 0%. The column was maintained at 40 °C.

### Animal experiments

Five-week-old male Wistar/ST rats (120–140 g) were purchased from Japan SLC (Shizuoka, Japan). A total of 12 rats were caged individually in a room, and housed at 22–24 °C and 40–70% relative humidity, with a 12 h light/dark cycle. The rats were allowed free access to a control diet (solid type of certified diet MF; Oriental Yeast, Tokyo, Japan) and drinking water for an acclimatization period of one week. All the animals were treated and cared for in accordance with the guidelines of the National Institutes of Health (NIH) for the use of experimental animals. All experimental procedures were approved by the Animal Care Committee of Kyoto Prefectural University (KPU280609). After the acclimatization period, the rats were divided into four groups (*n* = 3 for each group); control diet (C), control diet + pyroGlu-Leu (C + pEL), high fat diet (HF), and high fat diet + pyroGlu-Leu (HF + pEL). Rats in all groups received experimental diets for five weeks. The rats in the HF and HF + pEL groups were fed a solid type of high fat diet (60% kcal/total kcal; D12492, Research Diets, New Brunswick, NJ). The rats were allowed free access to either the control or high fat diet, as well as drinking water. PyroGlu-Leu was orally administered via drinking water. Drinking water containing pyroGlu-Leu (3.6–8.3 mg/L) was prepared every week to administer pyroGlu-Leu at 1.0 mg/kg body weight on the basis of consumption of drinking water in the previous week. The feces were collected from each rat on the final day and stored at −80 °C until the microbiota analysis. The rats were sacrificed after five weeks from beginning of the experiment by puncturing the inferior vena cava of the rats that were anesthetized using pentobarbital sodium (40–50 mg/kg). The small intestines were dissected. The inner content of intestines were flushed with 15 mL of physiological saline. The washed intestines (duodenum, ileum, and colon) and the inner contents of small intestines were collected and stored at –80 °C until use. Body weight were measured every two days and blood biochemical parameters (alanine aminotransferase; ALT, aspartate aminotransferase; AST, total cholesterol; TCHO, triglyceride; TG, HDL-cholesterol; HDL and LDL-cholesterol; LDL) were determined by outsourcing to Oriental Yeast (Tokyo, Japan).

### Microbiota analysis

The copies of e two major phyla of rat microbiota, *Firmicutes* and *Bacteroidetes*, were evaluated by qPCR, as described previously.^9^ Briefly, DNA was extracted from 0.5 g of each feces using a QIAmp DNA StoolMini Kit (Qiagen, Venlo, Netherlands), according to the manufacturer’s instructions. qPCR analysis was outsourced to the Primary Cell Division of Cosmo Bio (Sapporo, Japan).

### Peptides extraction from the intestines

The duodenum, ileum, and colon were cut into small pieces using scissors. The pieces (100 mg) were homogenized with 100 μL of PBS in a BioMasher II (Nippi, Tokyo, Japan). The homogenates were further mixed with 200 μL of 60% acetic acid and homogenized again. These homogenates were centrifuged at 13,000 × *g* for 10 min and the supernatants were collected. This solvent (30% acetic acid) has been used to preferentially extract animal antimicrobial peptides.^23^

### Size exclusion chromatography (SEC)

The 30% acetic acid extracts of ileums were purified by passing them through Ultrafree-MC (pore size 5 µm; Merck, Darmstadt, Germany) packed with Sephadex G-25 (fine grade; GE Healthcare, Buckinghamshire, England). Samples were eluted by spinning the column at 815 × *g* for 1 min. The clarified samples (200 µL) were subjected to SEC using a Superdex peptide 10/300 GL (GE Healthcare) equilibrated with 0.1% formic acid containing 10% acetonitrile at a flow rate of 0.5 mL/min. Fractions were collected every 1 min.

### Liquid chromatography mass spectrometry (LC-MS)

**Fig. 4:**
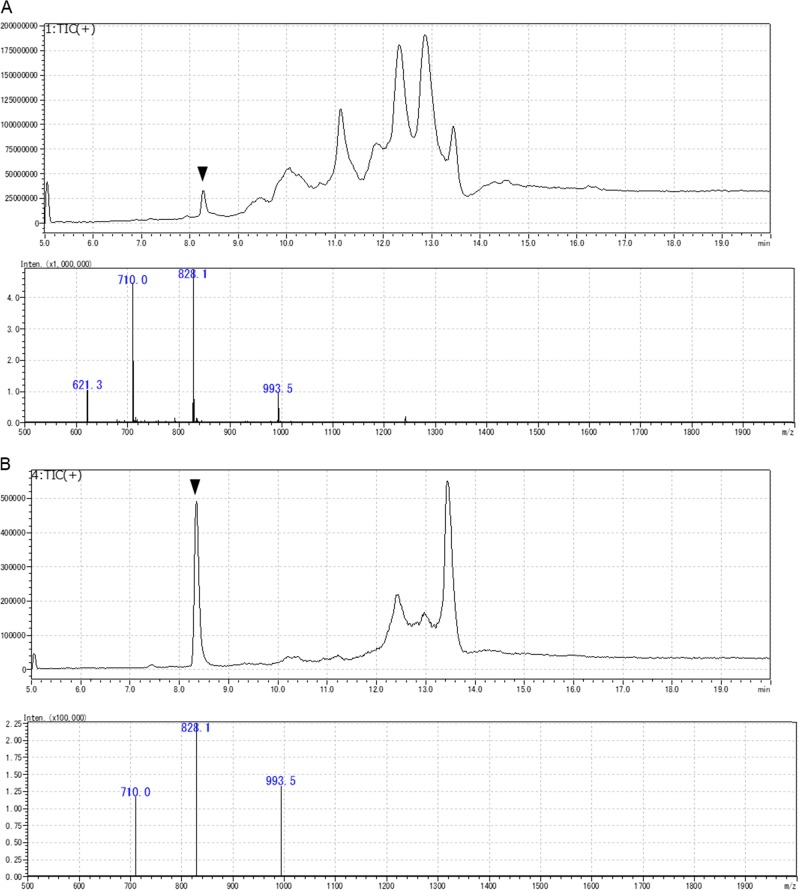
Mass chromatograms of RP-HPLC-MS of SEC Fr. 32 of HF + pEL group and mass spectra of the peak marked with arrowhead. Total ion monitoring mode **a** and selected (*m/z* = 710.0, 828.1, 993.5) ion monitoring mode **b**, respectively. The peptides whose molecular weight could be estimated were detected by LC-MS in SIM mode for the highest 2 or 3 multivalent ions. The *Y* axis represents ion intensity

The aliquots of SEC fractions 14–35 were clarified by passing them through a filter W (pore size 0.45 µm, 4 mm i.d.; Nacalai Tesque). The peptides in the SEC fractions (10 µL) were resolved by RP-HPLC using a Cosmosil Protein-R (5 µm, 2.0 mm i.d. ×150 mm; Nacalai Tesque). The column was equilibrated with 0.1% formic acid (solvent A). The elution was performed using a binary linear gradient of solvent A and 0.1% formic acid containing 80% acetonitrile (solvent B) at a flow rate of 0.2 mL/min. The gradient program was as follows: 0–10 min; B 0–50%, 10–10.1 min; B 50–100%, 10.1–20 min; B 100%, 20–20.1 min; B 100–0%, 20.1–30 min; B 0%. The column was maintained at 40 °C. The peptides were detected by ESI-MS using LCMS 8040 (Shimadzu, Kyoto, Japan) in total ion monitoring mode at positive ion mode. The molecular weights of the peptides in peaks were estimated on the basis of the *m/z* ratio of the multivalent ions. The *m/z* ratio of the adjacent multivalent ions were designated to *X*_*n*_ and *X*_*n*−1_, where *n* is the charge number. Assuming that all ions were generated by coupling with protons, the following simultaneous equations were formularized: *X*_*n*_ = (*Y* + *n*)/*n* and *X*_*n*−1_ = (*Y* + *n* − 1)/(*n* − 1), where *Y* is the molecular weight of the target peptide. The total ion chromatogram of SEC fraction 32 of the HF + pEL group is shown in Fig. 4 panel a (upper). The spectrum of the peak is denoted by an arrow in Fig. 4 panel a (lower). Multivalent ions, with an *m/z* of 621.4, 710.0, 828.1, and 993.5, were observed. All two adjacent ions were used for the estimation of molecular weights using the aforementioned equation. Consequently, the molecular weight was estimated to be 4962 Da, where the charges for the ions were 8, 7, 6, and 5, respectively. The peptides whose molecular weight could be estimated were detected by LC-MS in SIM mode for the highest 2 or 3 multivalent ions. For example, 3 ions (*m/z* = 710.0, 828.1, and 993.5) were selected for the peak marked with an arrow (Fig. 4 Panel a). The 30% acetic acid extracts were diluted with 100 times its volume of 0.1% formic acid containing 10% acetonitrile, and the diluents were then directly subjected to LC-MS analysis. The total ion chromatogram of SIM targeting *m/z* = 710, 828.1, and 993.5 is shown in Fig. 4 panel b (upper). If all the ions used for SIM were observed as shown in Fig. 4 panel b (lower), the peak area was recorded. In some cases, the molecular weights of the peptide was also evaluated by MALDI-TOFMS using an AXIMA Performance (Shimadzu). Sinapinic acid (10 mg/mL) was used as the matrix. Insulin and apomyoglobin were used for calibration. Peptides were purified by RP-HPLC using Protein-R by the same condition as described above. Aliquots of the peptide solution (5–20 ng/0.5 μL) and the matrix (0.5 μL) were added to the sample plate. The mass range was set from *m/z* 1000 to 20,000, the pulsed extraction was optimized at 7000 Da, the ion gate was set at 1000 Da, and the laser application power was started from 80.

### Peptide sequence

The amino acid sequences of the purified peptides were estimated by Edman degradation using a PPSQ-21 (Shimadzu). The amino acid sequences were assigned to protein using Basic Local Alignment Search Tool (BLAST).

### Detection of tryptic peptides of rattusin and defensin alpha 9

The presence of active form of rattusin and defensin alpha 9 in the inner contents of the small intestines was determined by detecting the tryptic peptides that are potentially derived from them. The suspension of the inner contents in PBS was centrifuged at 12000 × *g* for 10 min. The peptide concentration in the supernatant was evaluated by determining the absorbance at 280 nm using a Nanodrop Lite (Thermo Fisher Scientific, Waltham, MA). The concentration was adjusted to 1 mg/mL. The sample solution (48 μL) was mixed with 2 μL of 500 mM DTT and kept at 60 °C for 1 h. Thereafter, 4 μL of 500 mM 2-iodoacetamide (IAA) was added the solution and allowed to stand for 30 min in the dark. To terminate the alkylation process, 1 μL of 500 mM DTT was added. The alkylated peptides were digested with 1 μg of trypsin (1 μL) (MS grade, Thermo Fisher Scientific) at 37 °C for 24 h. The reaction was terminated by cooling at −30 °C. The contents of LR, VR, LSR, LEIR, and WPWK in the tryptic digests of the inner contents were evaluated by LC-MS/MS in MRM mode. The MRM conditions were optimized using synthetic peptides, which were pretreated with DTT and IAA, by LabSolutions Version 5.65. The tryptic digests were clarified by passing them through a filter, as described above, and then aliquots (10 µL) were injected into an Inertsil ODS-3 (5 µm, 2.1 mm i.d. ×150 mm; GL Sciences, Tokyo, Japan) equilibrated with 0.1% formic acid (solvent A). An elution was performed using a binary linear gradient of solvent A and 0.1% formic acid containing 80% acetonitrile (solvent B) at a flow rate of 0.2 mL/min. The gradient program was as follows; 0–15 min; B 0–50%, 15–20 min; B 50–100%, 20–25 min; B 100%, 25–25.1 min; B 100–0%, 25.1–35 min; B 0%. The column was maintained at 40 °C.

### Statistical analysis

All analyses were performed for every rat (*n* = 3). The results were presented as the mean ± standard deviations (SD). For microbiota analysis, body weight gain, blood parameters, and comparison of the effect of pyroGlu-Leu on rattusin propeptide between duodenum, ileum, and colon, the significant differences between the groups were evaluated by Tukey’s test. For other analyses, the significant differences between each diet (C vs. C + pEL or HF vs. HF + pEL) were evaluated by Student’s *t*-test. Differences of *p* < 0.05 were considered significant. Statistical analysis was performed using GraphPad Prism version 6.04 (USACO Corporation, Tokyo, Japan).

### Reporting summary

Further information on research design is available in the Nature Research Reporting Summary

## Supplementary information

Supplementary information Figures and legends.

Reporting summary

## Data Availability

All relevant data are available from the corresponding author upon request.

## Supplementary information

**Supplementary information** accompanies the paper on the *npj Science of Food* website (10.1038/s41538-019-0050-z).
