# Supplementary material for: Pyroglutamyl leucine, a peptide in fermented foods, attenuates dysbiosis by increasing host antimicrobial peptide
Source: NPJ Sci Food. 2019 Oct 7;3:18. doi: 10.1038/s41538-019-0050-z (PMC6779755; doi:10.1038/s41538-019-0050-z)
Supplement: Supplementary file 1 — Supplementary information Figures and legends. [file 41538_2019_50_MOESM1_ESM.pdf]

Supplementary information

Supplemental Figure 1. Total ion chromatogram of RP-HPLC-MS of SEC Fr. 14-35.

Peaks were numbered. The Y axis of the chromatogram represents ion intensity. Three lines represent chromatograms of 3 samples from same animal group. A; control group (C), B; control + pEL group (C + pEL), C; high fat group (HF), D; high fat + pEL group (HF + pEL)

Supplemental Figure 2. Effect of pyroGlu-Leu on peak area of peptides in the 30% acetic acid extract of ileum.

The peptides which did not significantly change after pyroGlu-Leu administration are listed (n=3).

Supplemental Figure 3. Confirmation of *m/z* of peptides in peak 20 using MALDI-TOF-MS.

m/z 4963.53 was observed.

Supplemental Figure 4. Comparison of effect of pyroGlu-Leu on contents of rattusin propeptide in 30% acetic acid extracts between duodenum, ileum, and colon.

The propeptide of rattusin in the organs was detected in LC-MS in SIM mode. Peak area was collected (n=3 for each tissue) and shown as % of that in ileum in control group. Different letters indicate significant differences ($p<0.05$) by Tukey's test.

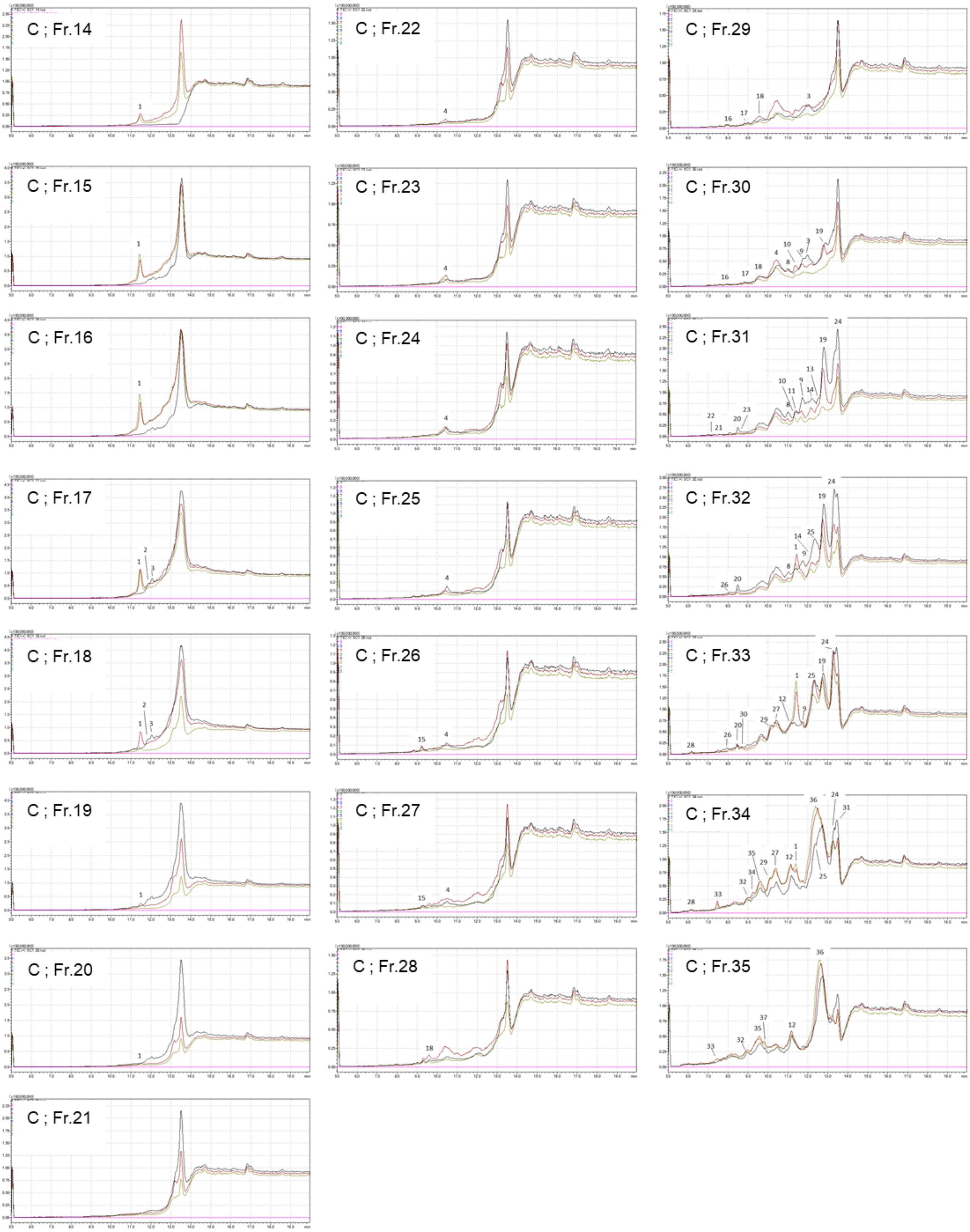

Supplemental Figure 1. (A)

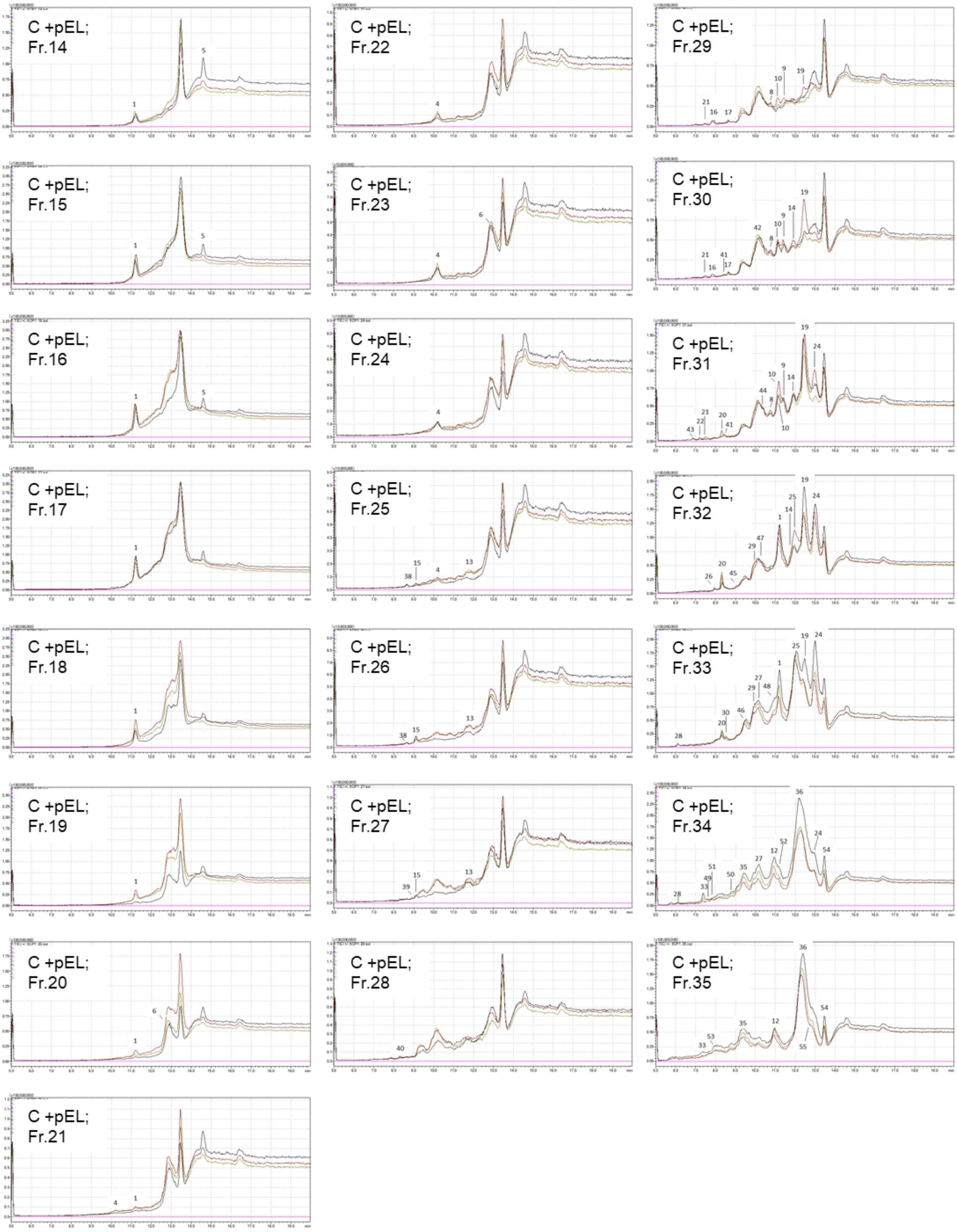

Supplemental Figure 1. (B)

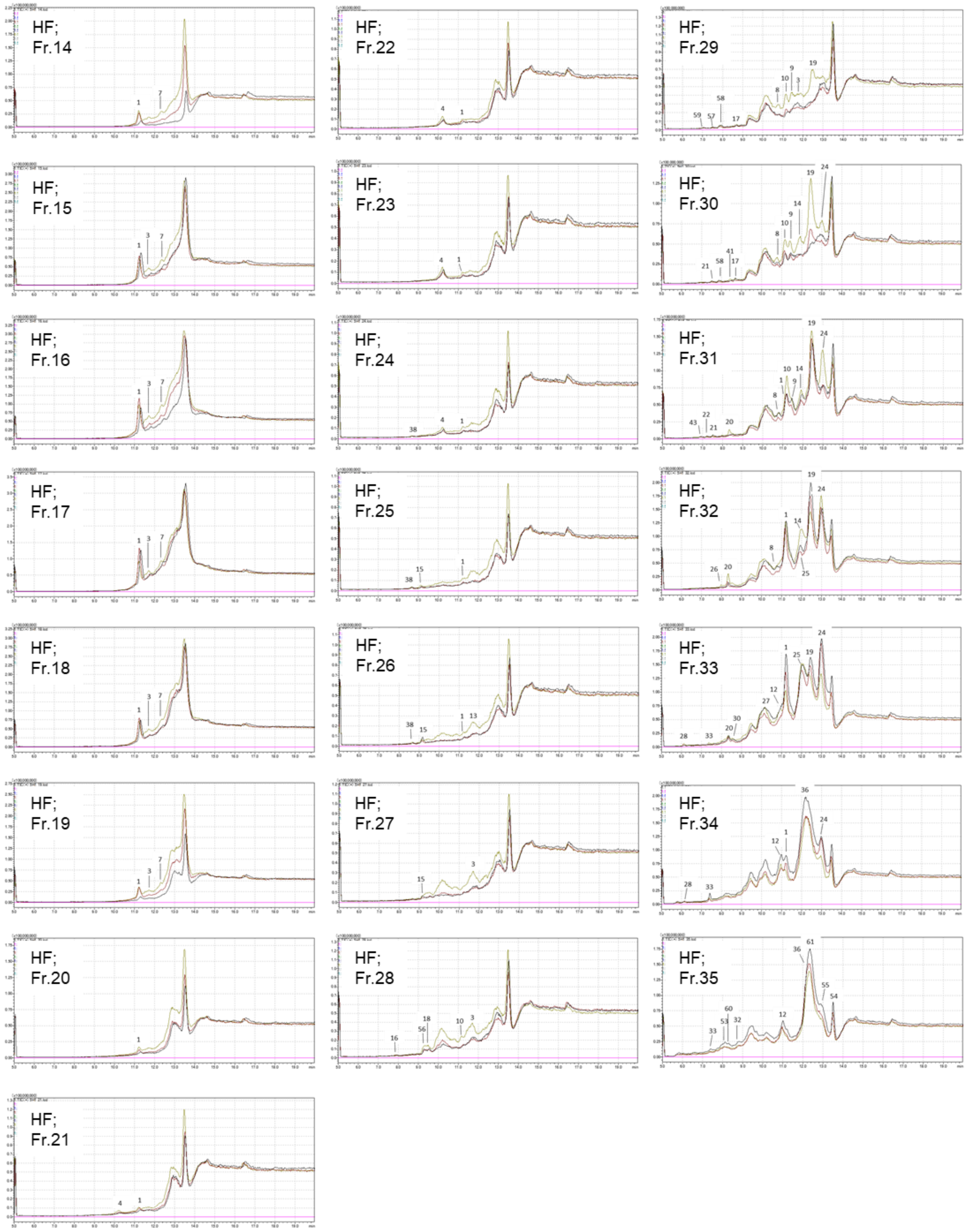

Supplemental Figure 1. (C)

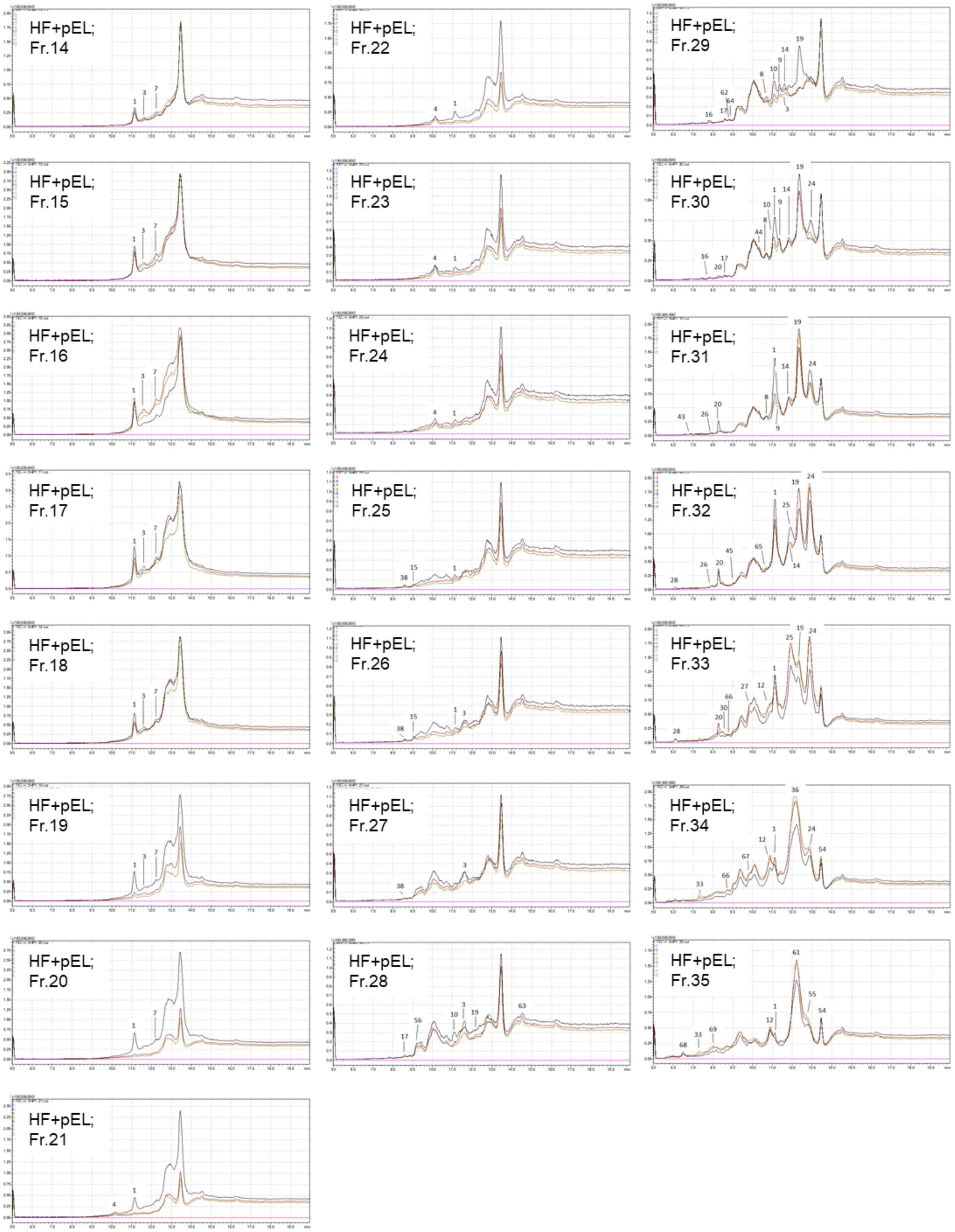

Supplemental Figure 1. (D)

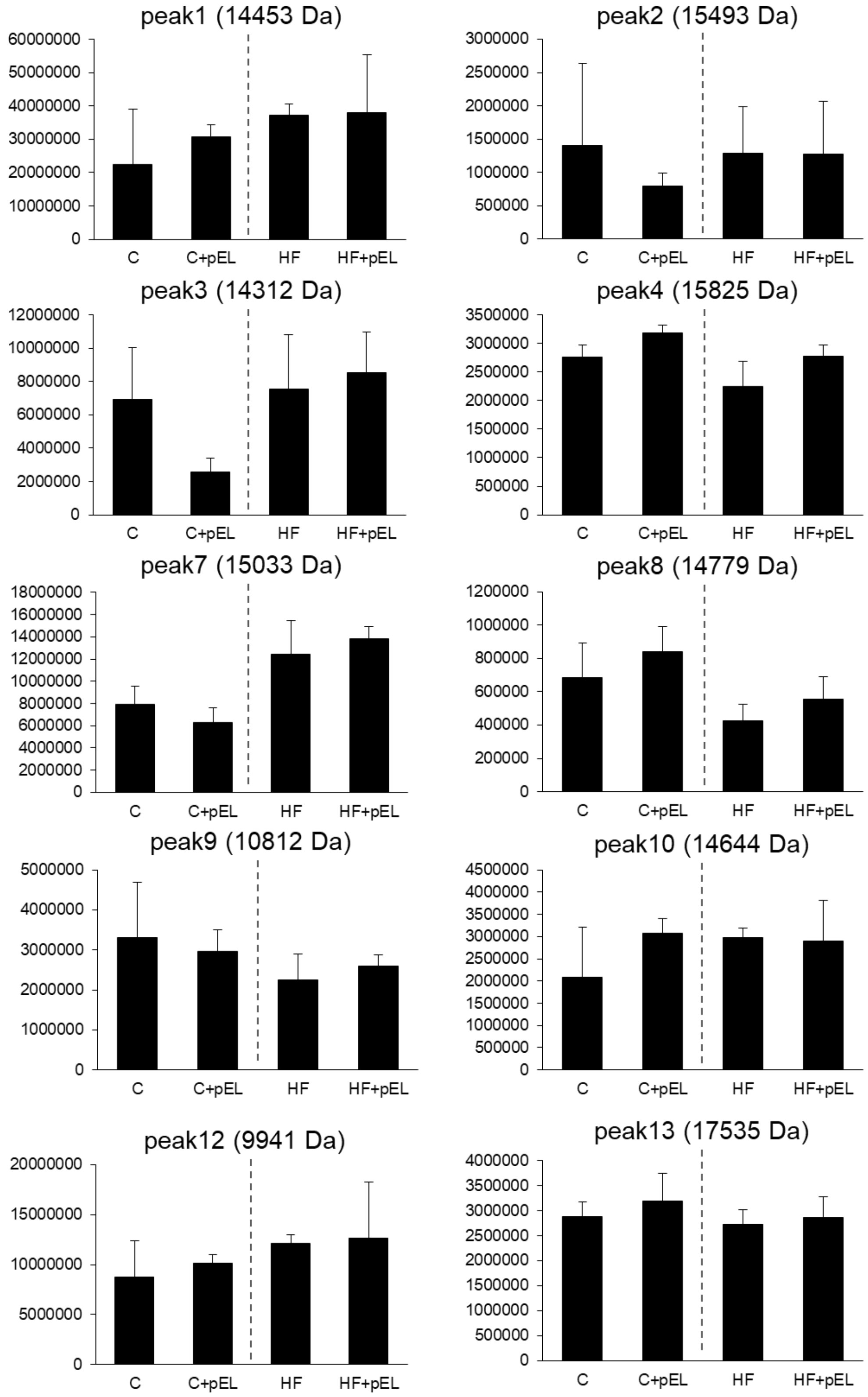

peak1 (14453 Da)
60000000
50000000
40000000
30000000
20000000
10000000
0
C
C+pEL
HF
HF+pEL
peak2 (15493 Da)
3000000
2500000
2000000
1500000
1000000
500000
0
C
C+pEL
HF
HF+pEL
peak3 (14312 Da)
12000000
10000000
8000000
6000000
4000000
2000000
0
C
C+pEL
HF
HF+pEL
peak4 (15825 Da)
3500000
3000000
2500000
2000000
1500000
1000000
500000
0
C
C+pEL
HF
HF+pEL
peak7 (15033 Da)
18000000
16000000
14000000
12000000
10000000
8000000
6000000
4000000
2000000
0
C
C+pEL
HF
HF+pEL
peak8 (14779 Da)
1200000
1000000
800000
600000
400000
200000
0
C
C+pEL
HF
HF+pEL
peak9 (10812 Da)
5000000
4000000
3000000
2000000
1000000
0
C
C+pEL
HF
HF+pEL
peak10 (14644 Da)
4500000
4000000
3500000
3000000
2500000
2000000
1500000
1000000
500000
0
C
C+pEL
HF
HF+pEL
peak12 (9941 Da)
20000000
15000000
10000000
5000000
0
C
C+pEL
HF
HF+pEL
peak13 (17535 Da)
4000000
3500000
3000000
2500000
2000000
1500000
1000000
500000
0
C
C+pEL
HF
HF+pEL

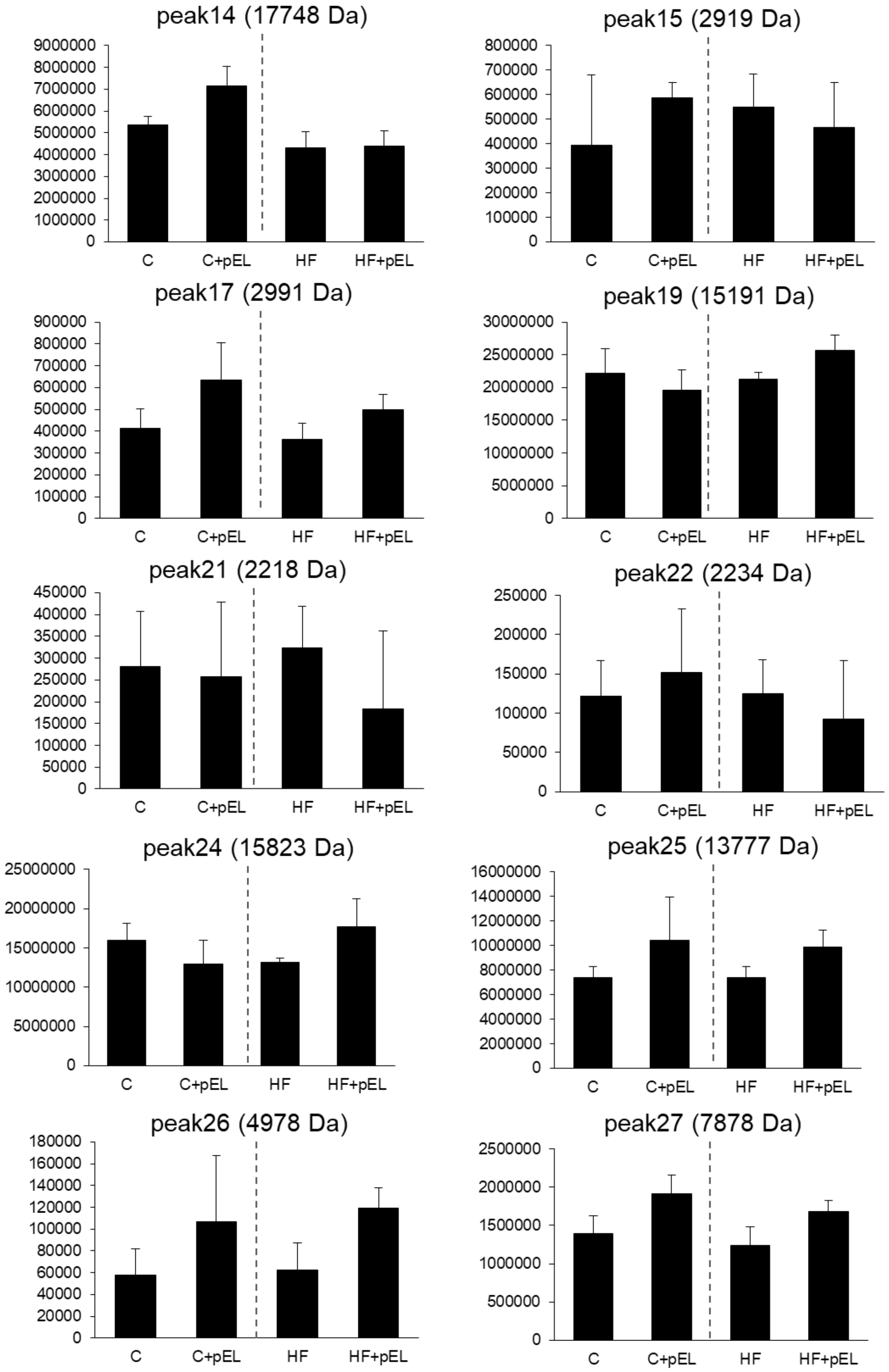
peak14 (17748 Da)
peak15 (2919 Da)
peak17 (2991 Da)
peak19 (15191 Da)
peak21 (2218 Da)
peak22 (2234 Da)
peak24 (15823 Da)
peak25 (13777 Da)
peak26 (4978 Da)
peak27 (7878 Da)
C
C+pEL
HF
HF+pEL

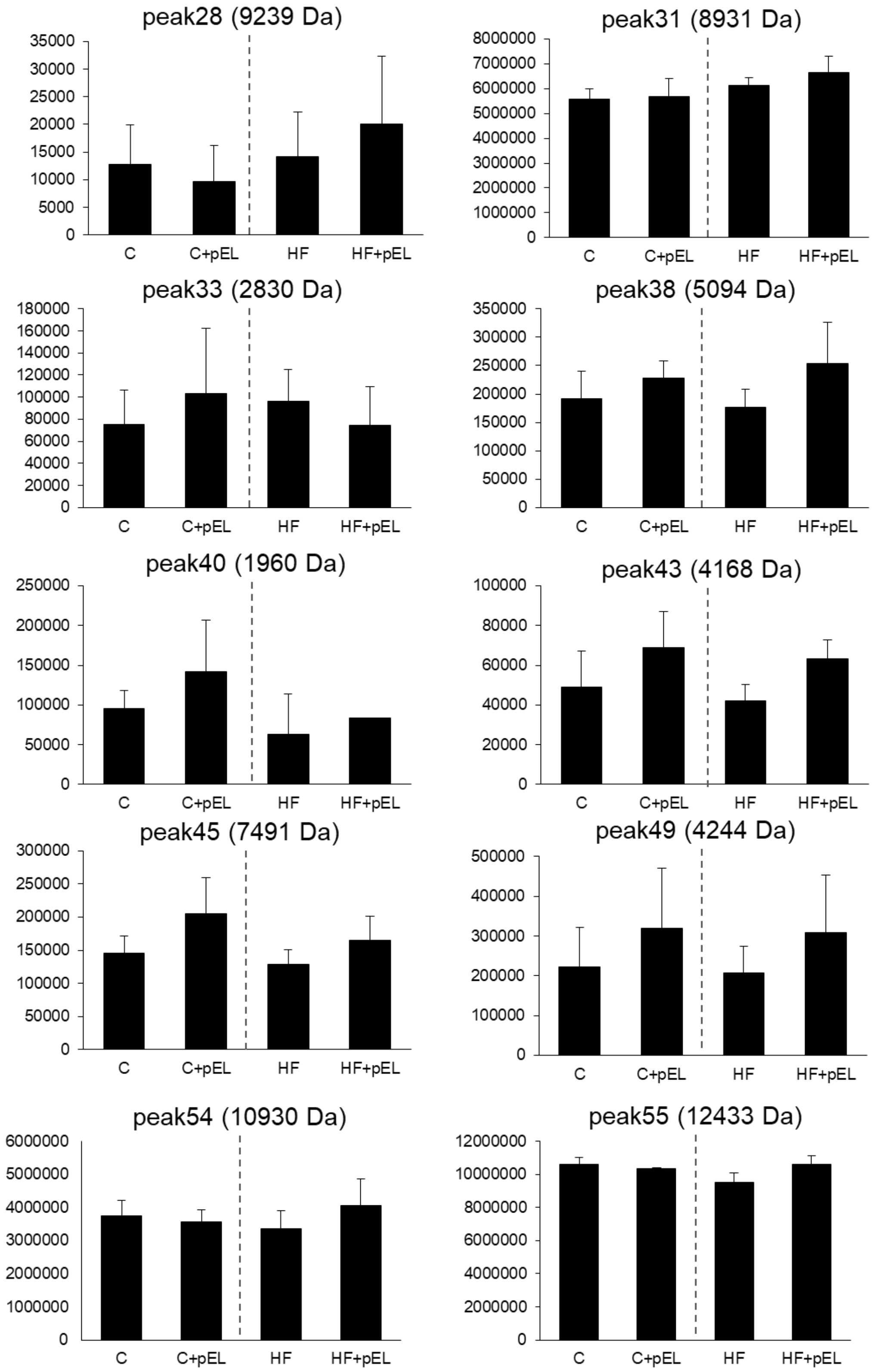
peak28 (9239 Da)
35000
30000
25000
20000
15000
10000
5000
0
C
C+pEL
HF
HF+pEL
peak31 (8931 Da)
8000000
7000000
6000000
5000000
4000000
3000000
2000000
1000000
0
C
C+pEL
HF
HF+pEL
peak33 (2830 Da)
180000
160000
140000
120000
100000
80000
60000
40000
20000
0
C
C+pEL
HF
HF+pEL
peak38 (5094 Da)
350000
300000
250000
200000
150000
100000
50000
0
C
C+pEL
HF
HF+pEL
peak40 (1960 Da)
250000
200000
150000
100000
50000
0
C
C+pEL
HF
HF+pEL
peak43 (4168 Da)
100000
80000
60000
40000
20000
0
C
C+pEL
HF
HF+pEL
peak45 (7491 Da)
300000
250000
200000
150000
100000
50000
0
C
C+pEL
HF
HF+pEL
peak49 (4244 Da)
500000
400000
300000
200000
100000
0
C
C+pEL
HF
HF+pEL
peak54 (10930 Da)
6000000
5000000
4000000
3000000
2000000
1000000
0
C
C+pEL
HF
HF+pEL
peak55 (12433 Da)
12000000
10000000
8000000
6000000
4000000
2000000
0
C
C+pEL
HF
HF+pEL

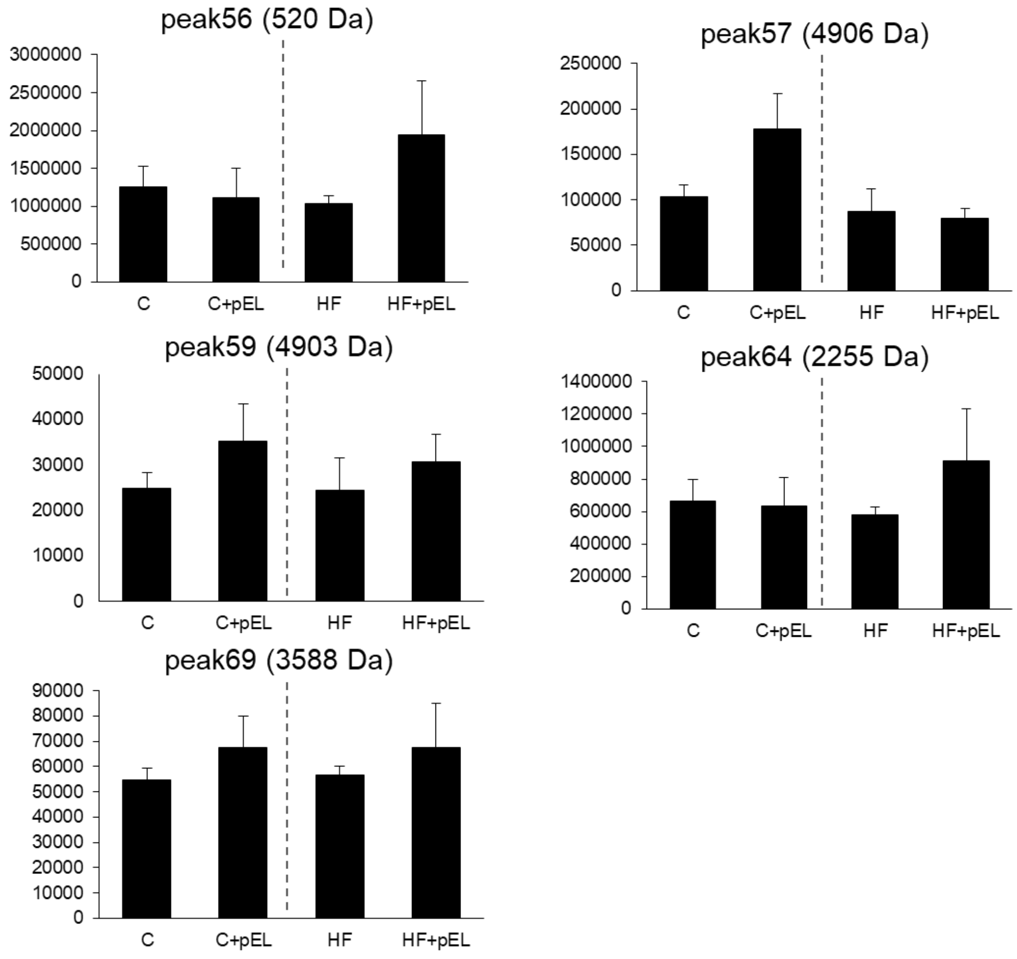

Supplemental Figure 2

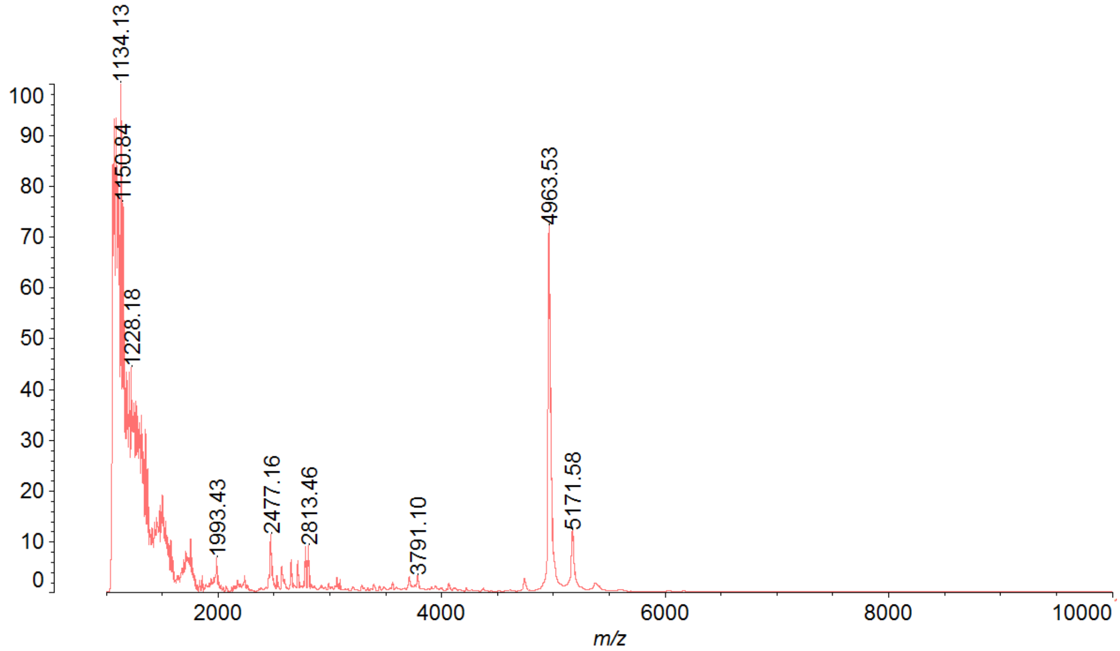

Supplemental Figure 3

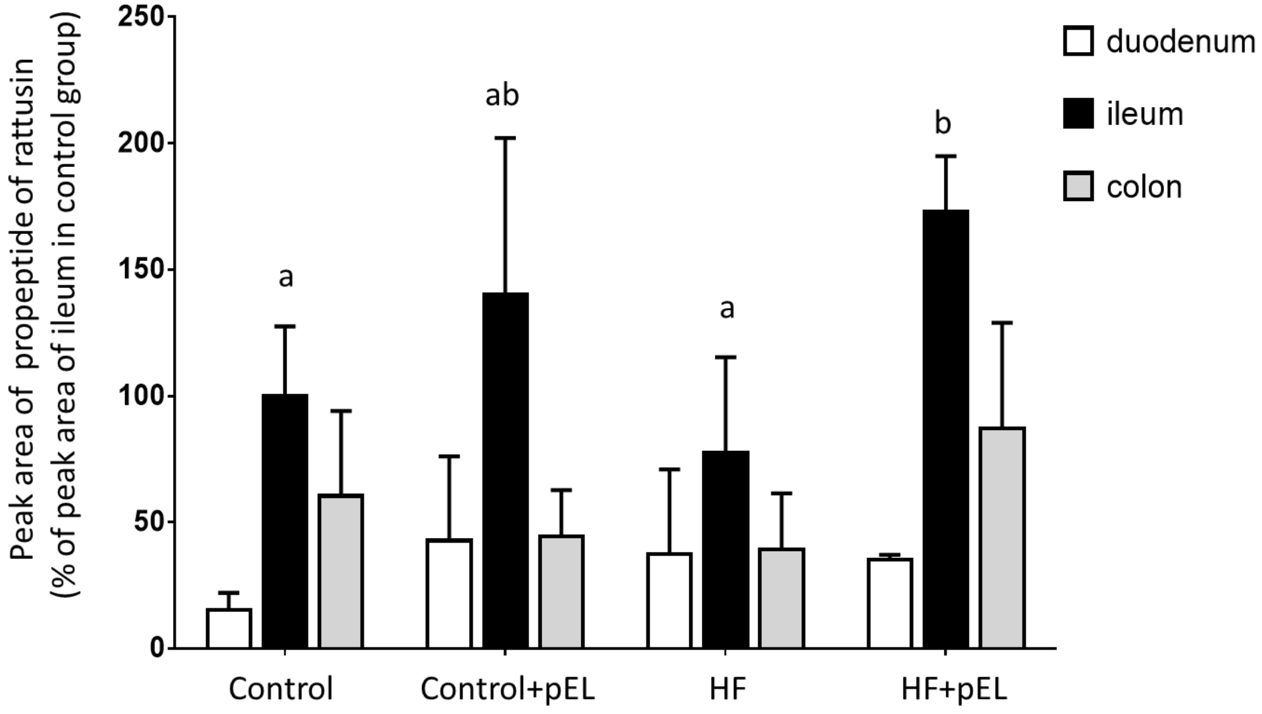

Supplemental Figure 4
